# Mercury Bioaccumulation in Benthic Invertebrates: From Riverine Sediments to Higher Trophic Levels

**DOI:** 10.3390/toxics9090197

**Published:** 2021-08-24

**Authors:** Laura Marziali, Claudio Roscioli, Lucia Valsecchi

**Affiliations:** CNR-IRSA Water Research Institute, National Research Council, Via del Mulino 19, 20861 Brugherio, MB, Italy; claudio.roscioli@irsa.cnr.it (C.R.); lucia.valsecchi@irsa.cnr.it (L.V.)

**Keywords:** methylmercury, aquatic insects, freshwater sediments, bioaccumulation

## Abstract

Riverine sediments are important sites of mercury methylation and benthic invertebrates may be indicators of Hg exposure to higher organisms. From 2014 to 2018, sediments and invertebrates were collected along a mercury gradient in the Toce River (Northern Italy) and analyzed for THg and MeHg. Concentrations in invertebrates, separated according to taxon and to Functional Feeding Group, ranged from 20 to 253 µg kg^−1^ dry weight (d.w.) for THg, increasing from grazers (*Leuctra*, *Baetis*, *Serratella*) to predators (*Perla*). MeHg ranged from 3 to 88 µg kg^−1^ d.w. in biota, representing 6–53% of THg, while in sediments it was mostly below LOD (0.7 µg kg^−1^), accounting for ≤3.8% of THg. The Biota-Sediment Accumulation Factor (BSAF, ranging 0.2–4.6) showed an inverse relation to exposure concentrations (THg in sediments, ranging 0.014–0.403 µg kg^−1^ d.w.) and to organic carbon. THg in invertebrates (up to 73 µg kg^−1^ wet weight), i.e., at the basal levels of the aquatic trophic chain, exceeded the European Environmental Quality Standard for biota (20 µg kg^−1^ w.w.), posing potential risks for top predators. Concentrations in adult insects were close to those in aquatic stages, proving active mercury transfer even to terrestrial food chains.

## 1. Introduction

Freshwater sediments are an essential reservoir where mercury accumulates, deriving from atmospheric deposition, terrestrial runoff, and local contamination sources [1,2,3]. Sediments can be important sites for methylation, which is favored under anaerobic conditions by the presence of sulphate- or iron-reducing bacteria at the water-sediment interface [4,5]. Methylmercury (MeHg) is the most toxic mercury compound, exerting neurotoxic effects, and it is easily released by sediments and bioaccumulated in food webs [2,6,7]. Moreover, MeHg shows a strong biomagnification potential, significantly increasing both as concentration and as a percentage to total mercury (THg) with increasing trophic level, posing the risk of secondary poisoning to top predators [8,9,10].

Mercury transfer from abiotic compartments (water, sediment) to biota is strongly influenced by site-specific physical and chemical factors, such as pH, redox conditions, presence of ligands, temperature, etc., which play an important role in Hg bioavailability [5,11,12]. In riverine ecosystems, these parameters may show large variations and the study of mercury cycling may become rather challenging. Mercury contamination is of high concern in freshwater ecosystems. In Europe, more than 45,000 water bodies fail to achieve the “good chemical status” according to Water Framework Directive because mercury concentrations exceed the Environmental Quality Standard (EQS) for water or, mostly, the EQS for biota (generally estimated on fish tissues) [13,14,15]. As well as this, fish consumption in more than 35% of US freshwaters is limited because of elevated MeHg values, even where no point sources of mercury are present [2].

Benthic invertebrates live in close proximity to sediments; thus, they are directly exposed to sediment contamination. These organisms are key vectors of mercury contamination from abiotic compartments to trophic chains [7]. They serve as food for many fish species in the aquatic habitat, while adult insects are also prey for terrestrial animals such as insects, birds, and bats [16]. However, studies on mercury bioaccumulation in freshwater invertebrates are limited (e.g., [10,11,17,18,19]). Aquatic invertebrates can be characterized by relevant concentrations of MeHg, mostly ranging between 10% and 90% of THg, determining significant transfer to predators [10,20]. Thus, they are important sentinels for mercury exposure for a wide range of higher organisms.

There is little information about the uptake routes and mechanisms responsible for mercury bioaccumulation in invertebrates [21,22]. However, it is widely accepted that diet is the dominant exposure pathway for mercury for most animals [2,12]. Aquatic insects belong to all categories of consumers, from herbivorous to predators, thus different Functional Feeding Groups (FFGs) may be characterized by different mercury concentrations and biomagnification within invertebrate communities may also occur [10,18]. Moreover, other determinants, such as specific physiological mechanisms, may influence the bioaccumulation potential of Hg [23]. For example, Cid et al. [16] found at the same site mercury concentrations in the mayfly *Ephoron virgo* two-fold higher in comparison to the caddisfly *Hydropsyche exocellata*, even if both are considered filter feeders. Moreover, the same authors highlighted that Hg concentrations in aquatic insects also change with size and life cycle stages. Notably, some works reported similar mercury values in aquatic and adult stages of freshwater insects, proving the efficient transfer of the contaminant to terrestrial ecosystems and to higher trophic levels [16,24,25,26].

Our aim was to investigate THg and MeHg concentrations in different invertebrate taxa collected in the Toce River (Northern Italy), characterized by a legacy mercury contamination deriving from a chlor-alkali plant. Organisms were grouped in different FFGs to test potential differences bound to trophic resources. The relation between bioaccumulation and sediment contamination, considered as primary mercury storage in the ecosystem, was analyzed. THg bioaccumulation in different life stages (pre-imaginal stages, exuviae, and adults) of some taxa was compared, to analyze the efficiency of mercury cycling in the environment of these organisms.

## 2. Materials and Methods

### 2.1. Study Area

**Figure 1:**
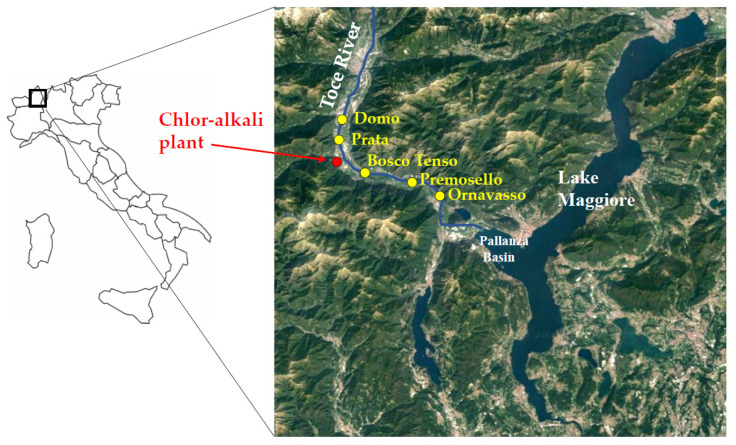
Map of the study area. Sampling points and the location of the chlor-alkali plant along the Toce River (Piedmont Region, Northern Italy) are reported.

The Toce River flows in the Ossola Valley, Central-Western Alps, Piedmont Region, Northern Italy. The river is 84 km long and is one of the main tributaries of Lake Maggiore, flowing into the Pallanza Basin with an average annual flow of 62 m^3^ s^−1^ (Figure 1). A mercury-cell chlor-alkali plant is located about 20 km upstream from the river mouth, in Pieve Vergonte town (VB), and it was definitively closed at the end of 2017. In the last century, until 1996, wastewaters were discharged directly into the Toce River, where mercury was proved to accumulate in sediments and biota. Peak concentrations of THg in sediments in the Pallanza Basin reached values up to 26 mg kg^−1^ dry weight (d.w.) in the 1940s, as estimated from the analysis of Hg vertical profiles in sediment cores sampled in the Pallanza Basin [27,28,29]. Mercury concentrations in the river sediments are constantly monitored by the International Commission for the Protection of the Italian-Swiss Waters (CIPAIS; www.cipais.org (accessed on 19 May 2021). Values downstream of the chlor-alkali plant are generally below or in line with the consensus-based Threshold Effect Concentration of 0.18 mg kg^−1^ d.w. (cb-TEC), i.e., the concentration below with toxic effects for benthic invertebrates are not expected [30], showing a mean value of 0.18 ± 0.20 mg kg^−1^ d.w. at the river mouth between 2008 and 2018 [29]. However, preliminary data on bioaccumulation in benthic invertebrates proved that THg values are generally above the European EQS for biota of 20 µg kg^−1^ wet weight (w.w.), showing the bioavailability of the contaminant and posing a potential risk of secondary poisoning to higher trophic levels [26,31]. Up to date, mercury concentrations in the river water are below the European EQS of 70 ng L^−1^ as a maximum allowable concentration [31].

Five sampling sites were selected along the 30 km-long final stretch of the Toce River (Figure 1): Domo and Prata are located 8.6 and 3.4 km upstream of the chlor-alkali plant, respectively, while Bosco Tenso, Premosello, and Ornavasso are located downstream the industrial area, at 3.7, 8.7 and 13.1 km downstream, respectively. This river stretch is relatively uniform for hydromorphological characters and hosts species-rich benthic communities both upstream and downstream of the industrial site [32]. The width of the riverbed is 40–60 m and the maximum depth is more than 1.5 m. In pool areas, predominant substrates are sand and gravel [32]. A few data on THg concentrations in porewater and at the water-sediment interface were derived in 2014–2015 using Diffusive Gradients in Thin films passive samplers [26,31] and resulted comprised between 3 and 56 ng L^−1^, with an increasing trend from Domo to Ornavasso.

### 2.2. Sediment and Benthic Invertebrate Sampling

At each sampling station, benthic invertebrates and sediments were collected in 2014, 2016, 2017, and 2018 in April and October, i.e., in two periods of intense emergence for aquatic insects and of intermediate discharge values of the nival-glacial flow regime.

Benthic organisms were extensively sampled using hand nets in the depositional areas of each site (i.e., in the pools, characterized by the maximum accumulation of the finest fractions of sediments such as silt and clay, where contaminants are mainly adsorbed), to collect appropriate biomass for chemical analysis. Invertebrate drift was limited to some small-sized individuals, as we observed using drift nets positioned during the samplings, thus we supposed that our samples were representative of the site. Benthic organisms were separated on-site in taxonomic groups (generally family) and were left in the river water for at least four hours to allow gut purging. Organisms of similar size were selected. Then they were sieved, gently dried with absorbent paper, and frozen at −18 °C in a portable freezer. In the laboratory, whole bodies were freeze-dried (72 h at 0.1 mbar and −45 °C; Telstar LyoQuest), homogenized with a ball mill (Retsch MM2000, Retsch Technology GmbH, Haan, Germany), and preserved in dark-glass bottles until mercury analysis.

Some specimens were preserved in 70% ethanolic solution and used for a more precise taxonomic identification under a stereomicroscope using identification keys [33,34,35,36]. The full list of taxa considered for chemical analysis is reported in Table 1. The most abundant taxa, which were generally present in all samples, were: Crustacea Gammaridae *Echinogammarus*, Diptera Tipulidae, Tabanidae, and Limoniidae, Ephemeroptera Heptageniidae *Ecdyonurus,* and Ephemeroptera Baetidae *Baetis*. The other taxa were not always present with sufficient biomass to carry out chemical analysis. The occurrence of each taxon at each site is reported in Figure S1 (Supplementary Material). The number of individuals per taxon per each sample ranged from 5 for *Perla* to over 200 individuals for *Baetis*, depending on the size and abundance of the organisms collected. A minimum of 0.05 g d.w. was necessary for THg analysis. However, for the four dominant taxa, a minimum of 0.5 g d.w. was generally collected.

Occasionally, it was possible to collect different life stages of the same taxon at the same site and sampling date. Pupae (about 10 individuals) were sorted from hand-net samples. Pupal exuviae (minimum 100 individuals) were collected directly from the surface of river boulders (e.g., Rhyacophilidae) or using drift nets (e.g., Heptageniidae). Adults (minimum 10 individuals) were picked with tweezers directly from the water surface. All were frozen, freeze-dried, and homogenized, as described above.

For sediments, at each site, different sub-samples were collected in the pool areas with a stainless-steel spoon and mixed in order to obtain a 2 L representative sample. Sediments were preserved in acid-washed dark-glass bottles at 4 °C until freeze-drying (72 h at 0.1 mbar and −45 °C). Sediments were sieved to separate the finest fraction (<63 µm grain size), which was preserved in dark-glass bottles and used for mercury analysis. Contaminants are frequently associated with the fine fraction of aquatic sediments [37], thus analysis of the <63 µm fraction is the most widespread in monitoring to reduce the variability between samples due to grain size composition [38]. Moreover, only the finest sediments can be ingested by small-sized invertebrate taxa (e.g., [39]).

**Table 1:** Invertebrate taxa collected during the study, taxonomic identification, main feeding habits (in brackets, secondary feeding habits are reported) according to literature [40] and Functional Feeding Groups (FFG) used to group the organisms for statistical analysis.

Order	Family	Genus/Species	Feeding Habits	FFG
Ephemeroptera	Ephemerellidae	*Serratella ignita*	grazer/gatherer	grazers
Ephemeroptera	Baetidae	*Baetis rhodani + Baetis alpinus/lutheri* gr.	grazer/gatherer	
Plecoptera	Leuctridae	*Leuctra* sp.	grazer/gatherer/shredder	
Ephemeroptera	Heptageniidae	*Ecdyonurus venosus*	grazer/gatherer/omnivore ^a^	gatherers-predators
Plecoptera	Perlodidae	*Dictyogenus + Isoperla*	predator (grazer/gatherer/shredder)	
Diptera	Limoniidae	*Hexatoma* sp.	predator (shredder)	
Diptera	Tipulidae ^b^	*Tipula lateralis*	gatherer (shredder)	
Diptera	Tabanidae ^b^	*Chrysops* sp. + *Haematopota* sp.	predator (gatherer)	
Trichoptera	Limnephilidae ^b^	*Allogamus* sp.	shredder (grazer/predator)	shredders
Amphipoda	Gammaridae	*Echinogammarus* sp.	shredder	
Ephemeroptera	Ephemeridae	*Ephemera danica*	active filter feeder (gatherer)	active filter feeders
Oligochaeta	Lumbricidae	*Eisenia tetraedra*	gatherer	gatherers
Trichoptera	Rhyacophilidae	*Rhyacophila* sp.	predator	small sized predators
Plecoptera	Perlidae	*Perla* sp.	predator (grazer)	large sized predators

^a^ according to literature, Heptageniidae show a tendency toward omnivory [41]. ^b^ the feeding habits of Diptera were derived from [34].

### 2.3. THg and MeHg Analysis

Total concentrations of mercury in organisms (analyzed in aliquots of 0.025 g d.w. of whole bodies) and sediments (in aliquots of 0.1 g d.w. of <63 µm grain size fraction) were determined by thermal decomposition, amalgamation, and atomic absorption spectrometry according to the US-EPA 7473 method [42] using an Automated Mercury Analyzer (AMA-254, FKV srl, Bergamo, Italy). The instrument sensitivity is 0.01 ng Hg, so the LOD calculated considering a sample weight of 0.025 g is 0.4 µg Hg kg^−1^. The Limit of Quantification (LOQ), calculated as ten times the standard deviation of the blank and considering the sample mass of 0.025 g, is 0.009 mg kg^−1^. The absolute instrument working range is 0.05 to 600 ng Hg.

For quality assurance, the certified reference materials BCR-CRM278 Mussel tissue (certified value = 0.196 ± 0.007 mg kg^−1^) and BCR-CRM320R Channel sediment powder (certified value = 0.85 ± 0.09 mg kg^−1^) of the Institute for Reference Materials and Measurements (IRRM, Geel, Belgium) were analyzed. Mean recovery was 105 ± 3.6% (n = 27) and 99 ± 1.5% (n = 16), respectively. Analyses were run in triplicate for sediments and in duplicate or triplicate for organism tissues, according to availability of biomass. The coefficient of variation was ≤5%.

MeHg was analyzed in five taxa (*Echinogammarus*, Diptera, *Ecdyonurus*, *Baetis,* and Trichoptera Limnephilidae) and in sediments collected at the five sites in April 2017. The analysis was performed by static headspace and GC-MS acquisition, whereas quantification was carried out using stable isotope dilution analysis. First, 0.25 g d.w. of homogenized tissue or 0.5 g d.w. of sediments were spiked with a 1 mL of ^201^Hg enriched solution diluted 1:100 (^201^Hg, 5.49 ± 0.04 µg g^−1^, 96.5%, ISC Science, Oviedo, Asturias, Spain). Then, they were microwave digested at 70 °C for 3 min adding 3 mL of HCl. Acid extracts were filtered (CA 0.4 µm) and brought to a pH of 5.5 adding 10 mL of buffer solution (sodium acetate/acetic acid 1 M) and 3 mL of 1 M KOH. MeHg ethylation was obtained by mixing each sample with 1 mL of NaBEt_4_ 1% in HPLC grade water (sodium tetraethylborate, 97%) directly in the pH-adjusted extract. GC-MS analysis was carried out using Thermo Fisher GC-MS system (respectively Focus GC and DSQ™ II single quadrupole) equipped with TriPlus RSH™ autosampler (Thermo Fisher Scientific, Rodano, Milan, Italy), able to static headspace technique. The samples were incubated for 12 min at 90 °C and 1.2 mL of the headspace was injected in the GC-MS. Ions considered for quantification were MeHg 215 + 244 m/z and Me^201^Hg 216 + 245 m/z. A stock standard solution was prepared by dissolving methylmercury chloride salt in a solution made with methanol and hydrochloric acid 18% with a proportion of 30/70% *v*/*v*. This solution was used to build a six-point calibration curve (0.03–2 µg MeHg). Cross contribution signal due to isotope pattern of native Hg and internal standard was corrected by software (Xcalibur 1.4). For quality assurance, the certified reference materials SRM-2974a *Mytilus edulis* tissue (National Institute of Standards and Technologies-NIST, Gaithersburg, MD, USA; reference value = 69.06 ± 0.81 µg kg^−1^) and ERM-CC580 estuarine sediment (IRMM, Geel, Belgium; reference value = 75 ± 4 µg kg^−1^) were analyzed. Recoveries were 102.5 ± 6.8% (n = 6) and 94.5 ± 8.8% (n = 6), respectively. The LOD of the method is 0.7 µg kg^−1^.

Organic carbon content (OC) in sediments was determined in 0.5 g d.w. samples by back-titration after oxidation with potassium dichromate in the presence of sulphuric acid, following the Walkley-Black procedure [43]. The LOD of the method is 0.14% OC, the LOQ is 0.46% OC, calculated as 3.3 times the LOD value [44]. The coefficient of variation for triplicate analysis was <5%.

### 2.4. Data Analysis

Each taxon was assigned to a Functional Feeding Group according to ecological information stored in the freshwaterecology.info database [40]. For Diptera, the feeding habit of each taxon was derived from [34].

To evaluate the bioavailability of mercury in sediments, the Biota-Sediment Accumulation Factor (BSAF, adimensional) was calculated according to the following formula [45]:

(1) $$BSAF=\frac{C_{org}}{C_{sed}}$$

where *C_org_* is the tissue concentration (mg kg^−1^ d.w.), *C_sed_* is Hg concentration in sediments (mg kg^−1^ d.w.).

Pearson’s correlation coefficient was calculated between variables: concentrations of THg or MeHg in organisms and sediments, OC, and percent fine sediments (i.e., <63 µm grain size).

One-way Analysis of Variance (ANOVA) followed by Tukey’s post-hoc test was carried out to test differences of THg or MeHg concentrations among sites, taxa, or FFGs. Kruskall-Wallis ANOVA followed by Dunn’s post-hoc test was carried out to test differences of MeHg concentrations among taxa or FFGs. T-test was used to test differences of THg and MeHg concentrations between sampling seasons (April vs. October).

Comparison between THg concentrations in different life stages was carried out with a *t*-test for dependent samples.

Prior to the previous analyses, the normality of data was tested with the Kolmogorov-Smirnov test and homogeneity of variance with Levene’s test. In the case of deviances, log_10_(x) transformation was carried out.

All analyses were run with Statistica 8.0 (StatSoft Inc., Tulsa, OK, USA) and Past 4.05 [46] software.

## 3. Results and Discussion

### 3.1. THg in Sediments and Biota

Total mercury concentrations in sediments at the most upstream site Domo (mean 0.036 ± 0.023 mg kg^−1^ d.w.) were in line with the background value of 0.044 ± 0.026 mg kg^−1^ d.w. estimated for the Toce River [32], while concentrations at the sites downstream of the industrial area (Bosco Tenso, Premosello, and Ornavasso) significantly exceeded the basal level, with a mean of 0.096 ± 0.075 mg kg^−1^ d.w. (ANOVA, *p* < 0.05 in comparison to Domo) (Figure S1). Sediments at Prata, which is located about 3 km upstream of the chlor-alkali plant, showed a slight mercury enrichment, with a mean of 0.055 ± 0.033 mg kg^−1^ d.w., and concentrations did not differ significantly from those of both Domo and the downstream sites (ANOVA, *p* > 0.05 for post-hoc comparisons), probably due to past atmospheric transport of the contaminant from the industrial area [32].

THg concentrations in sediments were positively correlated to OC (r = 0.66, *p* < 0.05). This relation is reported also in other case studies (e.g., [5,12]) and confirms the high affinity between Hg and organic matter, which is a strong ligand for this metal due to the strong binding affinity between Hg and thiol (-SH) groups [1,12,47].

Values were in line with other case studies in riverine ecosystems, characterized by legacy contamination deriving from chlor-alkali plants. For example, concentrations between 0.050 and 0.076 mg kg^−1^ d.w. are reported for the Savannah River (USA) [10]. At those levels, adverse effects on benthic invertebrates are not expected, according to the LOEC of 0.93 mg kg^−1^ d.w. reported for the benthic midge *Chironomus riparius* by Chibunda et al. [48]. In fact, benthic invertebrate communities show high diversity in this stretch of the Toce River, and they are only slightly influenced by sediment contamination, which may be bound also to the co-presence of other toxicants, such as DDT and its degradation products, and arsenic [29,32].

Similarly to what was observed in sediments, concentrations of THg in benthic invertebrates collected at Domo (0.048 ± 0.014 mg kg^−1^ d.w.) were lower than those observed at Prata (0.096 ± 0.037 mg kg^−1^ d.w.), which, in turn, were lower than values at the sites downstream of the factory (0.126 ± 0.037 mg kg^−1^ d.w.) (ANOVA, *p* < 0.001) (Figure S1).

Concentrations were in line with those of invertebrates collected even in rivers not influenced by local active pollution sources. For example, Heptageniidae (range 0.038–0.165 mg kg^−1^ d.w.), Limnephilidae (0.038–0.165 mg kg^−1^ d.w.) and Diptera (0.038–0.165 mg kg^−1^ d.w.) showed concentrations similar to those of the same taxa collected in two forested basins in US (range 0.05–0.212, 0.01–0.063 and 0.03–0.146 mg kg^−1^ d.w., respectively) [41]. On the contrary, Žižek et al. [49] reported values in River Idrijca (Slovenia), influenced by recent mercury mining activities, reaching concentrations of tens mg kg^−1^ d.w. for invertebrates. However, in the Toce, higher values downstream of the industrial area in comparison to the upstream sites prove the presence of a legacy contamination.

By comparing different taxa, all organisms showed similar values of THg, except for Ephemeroptera *Baetis*, which accumulated significantly less than the other groups (Figure S2). At the sites downstream of the factory, differences between taxa were more marked, and Plecoptera *Perla* showed the highest values (Figure S2b).

**Figure 2:**
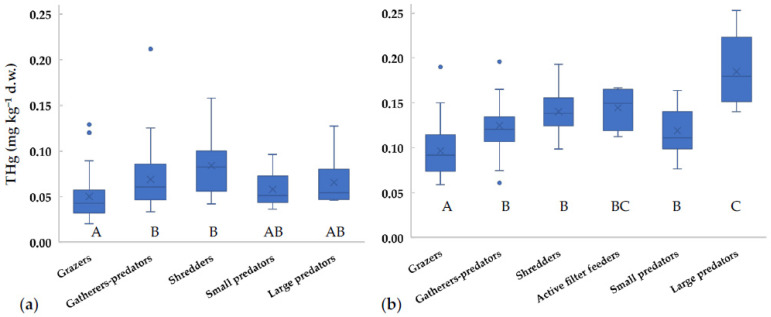
Concentrations of total mercury in different Functional Feeding Groups (FFGs) collected in the Toce River (**a**) upstream and (**b**) downstream of the industrial site between 2014 and 2018. x = mean value, horizontal line in boxes = median value, box = 25th–75th percentiles, whiskers = min-max range, points = outliers. Uppercase letters represent significant differences between FFGs according to ANOVA test followed by Tukey post-hoc (*p* < 0.05).

Since the diet is considered the main exposure route to mercury for aquatic organisms, THg values in different FFGs were compared (Figure 2). THg concentrations for most taxa did not significantly change with the season (*t*-test, *p* > 0.05), thus we supposed that FFG did not vary, and we could pool together data collected in different seasons. Similarly, Riva-Murray et al. [41] performed a stable isotope analysis in invertebrates of different US streams and found small variations between seasons. Our data showed THg values for grazers significantly lower than for the other groups (Figure 2). Gatherers-predators, shredders, and small-sized predators showed comparable values. At the downstream sites, large-sized predators showed concentrations significantly higher than those of the other FFGs, except for active filter feeders (Figure 2b). Similar results were obtained by Bates and Hall [18], who reported lower values for Gastropoda (grazers) in comparison to Dytiscidae/Notonectidae (predators) in US ponds. As expected, invertebrate FFGs generally show increasing amounts of THg with trophic levels [17,41].

Surprisingly, in the Toce River shredders showed concentrations in line with predators, while values are generally expected to be lower (e.g., [1,41]). However, this was observed also in other case studies, for example in the upper stream of the Francolì River (Spain), where Gammaridae (shredders) and Odonata (predators) showed similar values [50], as well as in Okefenokee Swamp (Georgia, USA) [51]. In fact, allochthonous inputs of terrestrial detritus and plant material may be an important mercury source to stream food webs [51,52]. A recent analysis of atmospheric mercury concentrations around the chlor-alkali plant shows that significant inputs are still deriving from the industrial area [53]. Thus, the mercury enrichment found in sediments at Prata, located upstream from the industrial plant, may derive from past and present contributions via atmospheric transport, even if the main wind direction is toward the river mouth.

### 3.2. MeHg in Sediments and Biota

MeHg in sediments was analyzed in samples collected in April 2017 and resulted below the LOD (0.7 µg kg^−1^), except for Bosco Tenso, where the concentration of 0.98 µg kg^−1^ d.w. accounted for 0.7% of THg. The highest value was found at Prata in 2013, where MeHg reached 3.8% (THg = 0.050 mg kg^−1^ d.w.) [31]. These percent values are in line with other studies on riverine sediments. The Savanna River (Georgia, USA), characterized by a legacy contamination deriving from a chlor-alkali plant, presented percent MeHg in sediments comprised between 1.1% and 2.5% [10]. MeHg percentage in the estuary of Penobscot River (Maine, USA), also impacted by a chlor-alkali plant, was 2.9 ± 0.3% [7]. Razavi et al. [54] reported for the Ontario River (Canada), downstream of a chlor-alkali plant, MeHg comprised between 0.02% and 0.6%, even if concentrations were significantly higher than those of the Toce (5 mg kg^−1^ d.w. for THg and 8.3 µg kg^−1^ d.w. for MeHg, on average). MeHg concentrations in the Idrija River sediments (Slovenia), which long received mercury related to cinnabar ore extraction activity, represented ≤0.067% of THg (up to 727 mg THg kg^−1^ d.w.) [49] and similar percent MeHg values were reported close to the mouth of the Isonzo River (Northern Italy) (THg up to 7.53 mg kg^−1^ d.w.), which receives water from the Idrija River [55].

MeHg concentrations in benthic invertebrates collected in 2017 ranged between 3 and 88 µg kg^−1^ d.w. and were correlated with THg in the organisms (r = 0.75, *p* < 0.05) and with THg in sediments (r = 0.77, *p* < 0.05). MeHg in tissues at sites upstream from the industrial area (mean of 16 ± 12 µg kg^−1^ d.w., n = 9) were significantly lower than concentrations in organisms collected downstream (51 ± 17 µg kg^−1^ d.w., n = 15) (*t*-test, *p* < 0.001). Besides, percent MeHg was lower in invertebrates collected upstream (21 ± 14%) in comparison to the downstream sites (40 ± 8%) (*t*-test, *p* < 0.001). Higher mercury bioavailability and/or methylation in sediments at the latter sites may explain this result.

**Figure 3:**
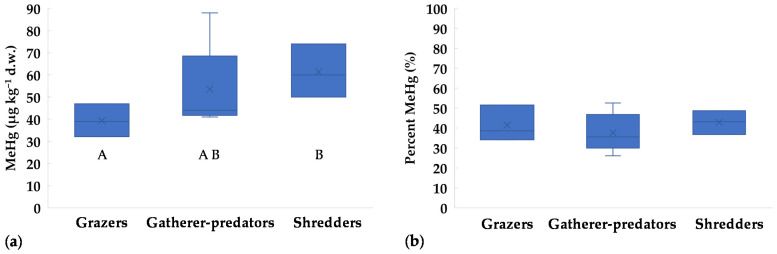
Concentrations of methylmercury in different Functional Feeding Groups (FFGs) collected in the Toce River downstream of the industrial site in 2017: (**a**) MeHg as µg kg^−1^ d.w.; (**b**) MeHg as percent relative to THg. x = mean value, horizontal line in boxes = median value, box = 25th–75th percentiles, whiskers = min-max range. Uppercase letters represent significant differences between FFGs according to Kruskall-Walis ANOVA test followed by Dunn’s post-hoc (*p* < 0.05).

Regarding FFGs, MeHg concentrations in grazers were lower than in shredders (Figure 3a). On the contrary, percent MeHg showed similar values in all groups, with maxima up to 53% of THg (Figure 3b). Low MeHg percentages (≤50%) were previously reported for grazing insects [10,18,20], while median values around 55% were found for Trichoptera Limnephilidae (shredders) [20]. For omnivores and predators, MeHg generally represents most of THg, e.g., >70% for Corixidae, Gerridae, Dytiscidae, Notonectidae, and crayfish [10,18,20]. In general, MeHg should increase with trophic level. This was observed by Mason et al. [17], who reported 20–40% MeHg for herbivores/detritivores and >70% for predators. Unfortunately, in our study the mass of large-size predators was not enough for MeHg analysis, thus a potential increase with trophic level could not be tested. However, this is not always observed in invertebrate communities, because the length of the trophic chain may be too low [41,56]. For example, Bates and Hall [18] found a δ^15^N increase of 1.44–1.99‰ from grazers to predators, which accounts for less than a trophic level (a minimum of 2‰ is generally accepted between trophic levels, [20]). Another drawback is that it may be difficult to establish a common δ^15^N baseline for different taxa. According to Diaz-Jaramillo et al. [56], MeHg at low trophic levels of benthic food webs can be better predicted by δ^13^C rather than δ^15^N signatures, showing the importance of detritus cycle and biofilm as a primary Hg exposure pathway to macroinvertebrates. Unfortunately, we did not perform stable isotope analysis, and this limits the analysis of the trophic relations between taxa and food sources.

### 3.3. Relation between BSAF and Environmental Variables

Tissue concentrations of THg (and MeHg) generally increased with increasing environmental concentrations, i.e., sediment THg concentrations, but the correlation coefficient between these variables was low (r = 0.23, *p* < 0.05). Similar results were obtained even considering single taxa, as the highest coefficient r was 0.36 (*p* < 0.05), obtained for Heptageniidae, and it was even not significant for most taxa. The relation was weak even normalizing THg values in sediments to percent OC (r = 0.33, *p* < 0.05 considering all data). This confirms that THg concentration in sediments cannot be used directly as a predictor of an organism’s accumulation [5,49]. This may be related primarily to geochemical factors and site-specific microbial activity, which affect mercury bioavailability and methylation rates. In rivers, only a small fraction of THg is generally bioavailable and it may change over time due to methylation/demethylation reactions, which are microbially mediated in sediments [4]. Thus, the local bioavailability of the metal is hardly predictable.

To evaluate bioavailability, the calculation of BSAF was carried out according to equation 1 for those taxa showing at least part of diet bound to sediments or particulate matter according to Table 1 (i.e., excluding the predators Rhyacophilidae and *Perla*). Values ranged between 0.2 and 4.6 (Figure S3). Mean values of each taxon were always above 1, showing efficient mercury bioaccumulation into living organisms (Figure S3) [12].

**Figure 4:**
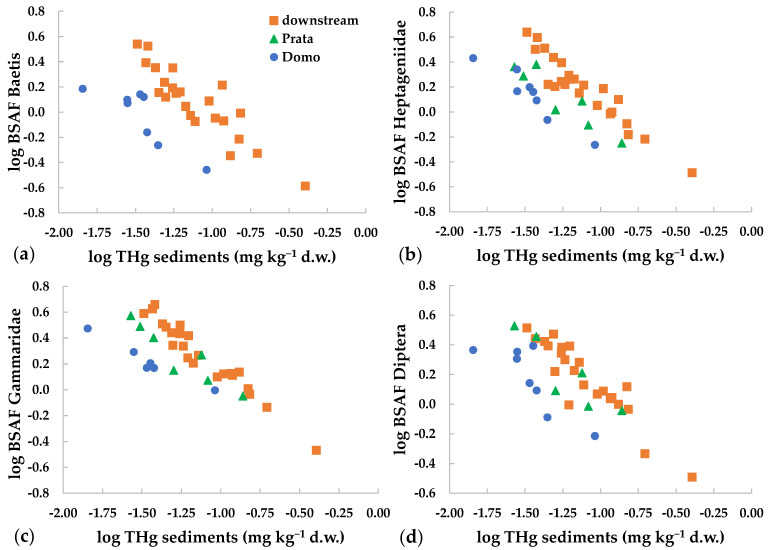
Relation between THg concentrations in sediments and BSAF values (log-transformed) for different invertebrate taxa collected at Domo, Prata and the three sites downstream of the industrial area (=downstream): (**a**) *Baetis*, (**b**) Heptageniidae, (**c**) Gammaridae, (**d**) Diptera.

An inverse relation was found between BSAF and THg in sediments (Figure 4). This showed that the bioaccumulation capacity was higher at lower environmental concentrations. This relation was observed for mercury and for several metals in the dissolved phase [21,23,57]. The inverse relation is known also between metal dietary concentrations and trophic transfer [8,19,57], while, to our knowledge, it is not reported in the literature for metal concentrations in sediments. This behavior has been related to the physiological regulation of uptake and excretion of metals, as well as to the storage and/or detoxification capacity of the organisms [19,57]. At low environmental concentrations, higher intake is observed for all metals, including mercury, since the uptake mechanisms for both essential and non-essential elements may follow similar routes [19]. At higher concentrations, internal regulation or saturation of ligand binding sites may occur, lowering the overall uptake rate of the contaminant [21].

It must be considered that in field studies the equilibrium between accumulation and exposure conditions may not be reached [23]. However, in our study, the inverse relation was observed for different taxa at different sites (Figure 4), reinforcing this finding. Notably, the inverse relation between THg concentrations in sediments or in the diet vs. mercury accumulated in the next highest trophic level may reduce concentration differences among sites and among trophic levels [19].

Other environmental parameters may drive mercury bioavailability. For instance, OC may decrease the bioavailability of mercury, and the inverse relationship between BSAF and OC was already observed [12]. Here, as well, the correlation between these variables is significant, even if values are highly dispersed (r = −0.46, *p* < 0.05, Figure S4a). On the contrary, relation with percent fine sediments was positive, even if weak (r = 0.23, *p* < 0.05, Figure S4b).

Other chemical and physical parameters may influence mercury bioavailability at the water-sediment interface, such as oxygen levels [12], as well as microbial activity [4], which may be highly variable in rivers. Moreover, it must be considered that we analyzed only sediments as the dominant route for mercury exposure, while porewater and bottom water may be other important pathways. For example, Xu et al. [10] were able to connect labile mercury concentrations in porewater to bioaccumulation in biofilm and in the higher levels of riverine trophic chains.

Unfortunately, MeHg analysis in sediments was hampered by the low values, which were generally below the LOD of our instruments. Therefore, we could not establish a relation between bioaccumulation and MeHg exposure or other environmental variables. Even if MeHg is recognized as the most bioaccumulative species of mercury, due to high bioavailability and low excretion rates, the relation between tissue concentrations and exposure is not so straightforward, as for THg, due to the complexity of physiological mechanisms which may drive accumulation and excretion, and in turn toxicity [21,22,23]. However, our data show that a significant part of mercury in the organism was in the organic form, raising the risk of biomagnification in the trophic chains.

### 3.4. Environmental Risk

According to the European legislation, the EQS for mercury in biota is 20 µg kg^−1^ w.w. of THg and applies to prey tissue with the aim to protect wildlife consumers of aquatic biota against secondary poisoning via the food chain [13,14]. The EQS was derived based on MeHg using the lowest available no observed effect concentration (NOEC) for birds and mammals and it is generally referred to fish, where the predominant form of mercury is MeHg (generally >80%) [13,14]. For comparison, by converting to wet weight mercury concentrations in invertebrates, values reached 73 µg kg^−1^ w.w. for THg and 17 µg kg^−1^ w.w. for MeHg (Figure S5). By considering THg, the EQS was exceeded in 44% of samples. It was never exceeded at Domo, while downstream of the factory the mean value was always above or in line with the EQS. By considering MeHg, the concentration of 20 µg kg^−1^ w.w. was almost reached by Heptageniidae and Gammaridae collected downstream.

Values of THg in invertebrates close to the EQS or even higher are frequently observed in freshwaters, even when point sources of contamination are not present [13,17,24]. Not surprisingly, values in insectivorous/omnivorous fish species collected in the Toce River [58,59] are one order of magnitude higher than the EQS (Figure S6). Many studies relate mercury concentrations in fish with their prey, such as benthic invertebrates [2,10,12]. The results obtained in the Toce River show how these organisms may actively transfer legacy contamination from sediments to higher trophic levels.

**Figure 5:**
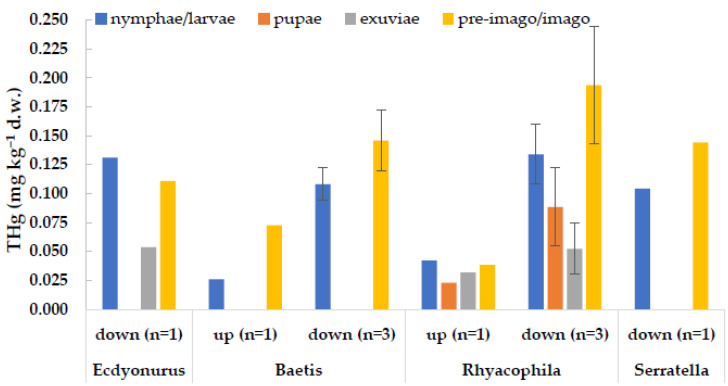
Concentrations of total mercury in different life stage of the same taxon, collected at the same site (up = upstream from the industrial area; down = downstream of the industrial area) and sampling date. Columns represent the mean value, bars are ±1 standard deviation. n = number of samples.

An interesting observation regarded the adult stages of some aquatic insects, which were captured during our surveys (Figure 5). Concentrations of THg in adults were generally in line with those measured in the corresponding larval stages (*t*-test for dependent samples, *p* > 0.05), proving an efficient transfer of the contaminant from the aquatic environment to terrestrial trophic chains. Thus, it seems that metamorphosis does not imply significant excretion of mercury. This is also supported by the low values observed in exuviae in comparison to those of larvae and adults (*p* < 0.05) (Figure 5). Similar results were obtained in a lab experiment with the dipteran *C. riparius* exposed to the Toce River sediments, which showed similar body burdens in larvae and in imagos [26].

As well, Cid et al. [16] reported similar concentrations between nymphs and adults of the mayfly *E. virgo* collected in mercury-contaminated river sites, while values in moults were significantly lower. Gimbert et al. [22] proved for *C. riparius* that exoskeleton, gut content, and cellular debris account only for 10% of Hg accumulated by the organism, while the cytosolic fraction represents most of the body burden (90%). This confirms that the main accumulation organ for mercury in freshwater insects is not the exoskeleton [21]. Similar concentrations of mercury in larvae and adults are reported also by Kraus et al. [25], as observed in case studies with low mercury concentrations. On the contrary, a higher fraction may be proportionally lost with metamorphosis when exposure concentrations increase [25], as reported by Rossaro et al. [60], who found in adults of *C. riparius* an average of 29% of THg concentration measured in larvae exposed to 5.5 µg L^−1^ HgCl_2_ solutions.

We did not analyze MeHg in adults. However, according to Chételat et al. [24] MeHg:THg ratio may even increase from larval to the adult stage: the authors reported MeHg concentrations 2.9 times higher in chironomid adults in comparison to larvae, representing the predominant form of mercury (82 ± 15% of THg). These results underline how aquatic insects may play a key role in mercury transfer not only to aquatic but also to terrestrial trophic chains, where adults are prey for other insects, amphibians, birds, and mammals like bats [16].

## 4. Conclusions

The Toce River represents a case study characterized by legacy contamination, where mercury bioaccumulation is still of concern, notwithstanding the relatively low concentrations in sediments.

The analysis of mercury in biota is a direct method for assessing bioavailability. Benthic invertebrates represent all categories of consumers, from herbivorous to secondary predators, giving the opportunity to analyze different exposure pathways and highlighting THg and MeHg concentrations at the base of aquatic and terrestrial food webs.

As expected, mercury values proved to increase from prey to predators, reaching concentrations above the EQS and thus potentially toxic to top predators. Results are consistent with previous studies, showing that mercury can cause secondary poisoning to wildlife at environmental concentrations not toxic to prey. The high persistence of the contaminant across insect metamorphosis, associated with low effects on larval and adult survival (as proved by the species-rich invertebrate communities of the Toce), may determine consistent flux and cycling in the environment.

Mercury concentrations, organic carbon, and the presence of fine-grained fractions proved to be significant variables determining bioaccumulation from sediments. However, other potential exposure routes, i.e., through porewater and bottom water, need to be investigated. Moreover, analysis of stable isotopes is necessary to improve knowledge on the trophic relations between different taxa and food sources.

## Data Availability

Data are available in the reports of the International Commission for the Protection of Italian-Swiss Waters (CIPAIS) regarding hazardous substances in Lake Maggiore (years 2014–2019), which are published and downloadable at www.cipais.org (accessed on 19 May 2021).

## Supplementary Materials

The following are available online at https://www.mdpi.com/article/10.3390/toxics9090197/s1, Figure S1: Concentrations of total mercury in sediments and in different taxa of benthic organisms collected in the Toce River, Figure S2: THg concentrations in different taxa collected in the Toce River upstream and downstream of the chlor-alkali plant between 2014 and 2018, Figure S3: BSAF values in different Functional Feeding Groups, Figure S4: Correlation between BSAF values and OC concentrations or percent fine sediments, Figure S5: Comparison of THg and MeHg concentrations (w.w.) in benthic invertebrates with the European EQS for biota, Figure S6: THg and MeHg concentrations in benthic invertebrates and fish collected in the Toce River.
