# Supplementary material for: Mercury Bioaccumulation in Benthic Invertebrates: From Riverine Sediments to Higher Trophic Levels
Source: Toxics. 2021 Aug 24;9(9):197. doi: 10.3390/toxics9090197 (PMC8473003; doi:10.3390/toxics9090197)
Supplement: Supplementary file 1 [file toxics-09-00197-s001.zip › toxics-1304909-supplementary.pdf]

toxics

MDPI

Article

Mercury Bioaccumulation in Benthic Invertebrates: From Riverine Sediments to Higher Trophic Levels

Laura Marziali *, Claudio Roscioli and Lucia Valsecchi

CNR-IRSA Water Research Institute, National Research Council, Via del Mulino 19, 20861 Brugherio, MB, Italy; claudio.roscioli@irsa.cnr.it (C.R.); lucia.valsecchi@irsa.cnr.it (L.V.)
* Correspondence: laura.marziali@irsa.cnr.it; Tel.: +39-039-216-942-07

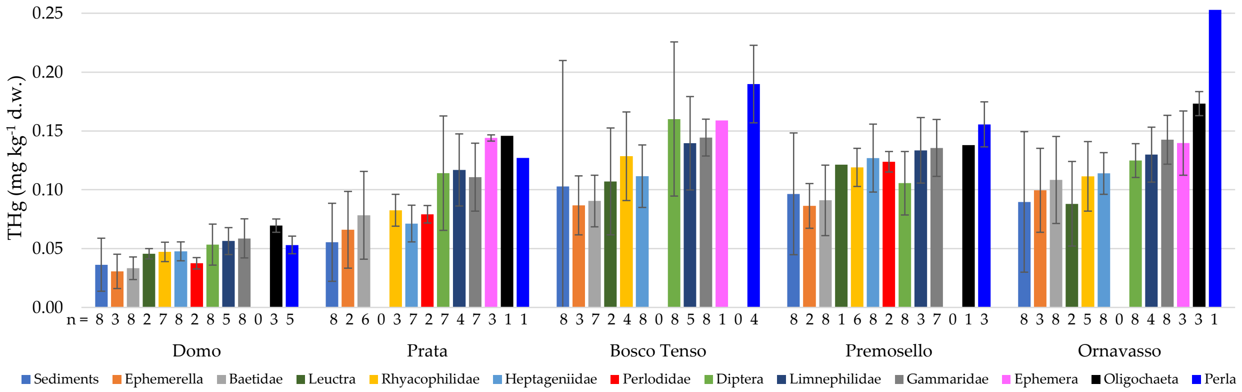

Figure S1. Concentrations of total mercury (THg) in sediments and benthic organisms (different taxa) collected at five stations in the Toce River (Northern Italy) between 2014 and 2018. Columns represent the mean value, bars are ±1 standard deviation, n (along the x axis) = number of samples (each sample is composed by a pool of specimens weighting at least 0.05 g d.w., to allow THg analysis).

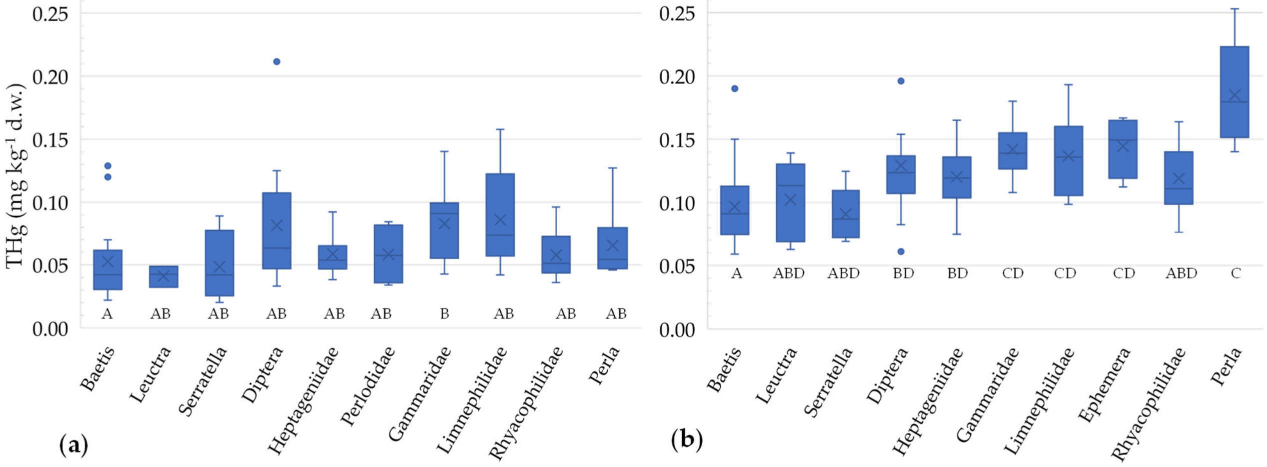

Figure S2. Concentrations of total mercury in different taxa collected in the Toce River (a) upstream and (b) downstream of the industrial site between 2014 and 2018. x = mean value, horizontal line in boxes = median value, box = 25th-75th percentiles, whiskers = min-max range, points = outliers. Uppercase letters represent significant differences between taxa according to ANOVA test followed by Tukey post-hoc ($p < 0.05$).

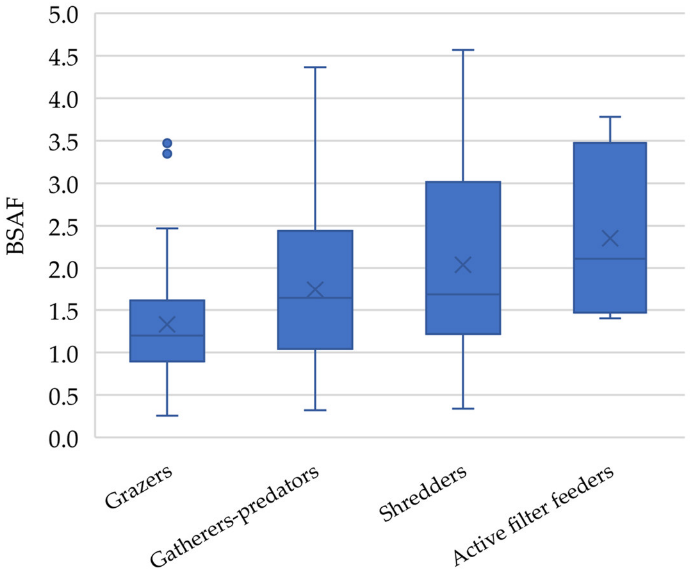

Figure S3. Box plot of BSAF values in different Functional Feeding Groups of benthic invertebrates collected in the Toce River downstream of the chlor-alkali plant.

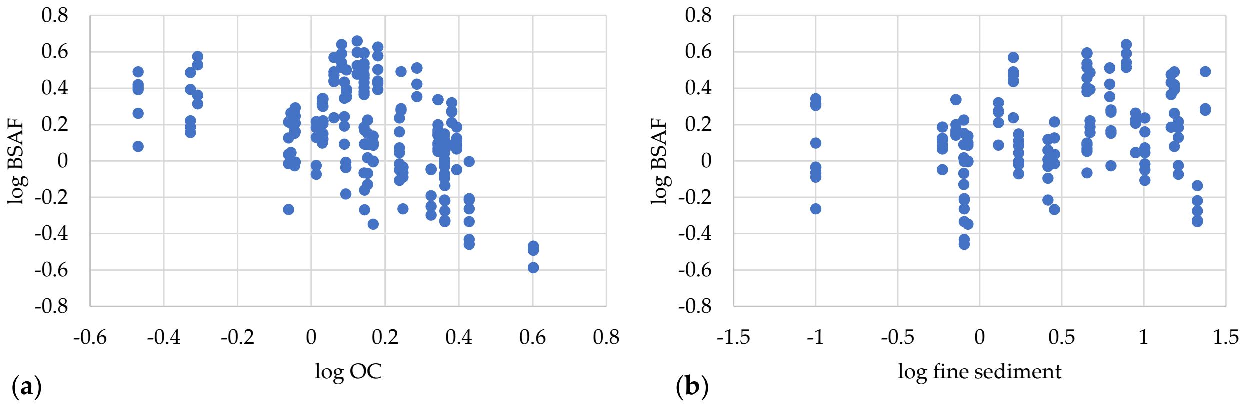

Figure S4. Pearson's correlation between log-transformed BSAF values (all taxa) and (a) OC concentrations in sediments ($r = -0.46$, $p < 0.05$) and (b) percent fine sediments ($r = 0.23$, $p < 0.05$), considering all dataset.

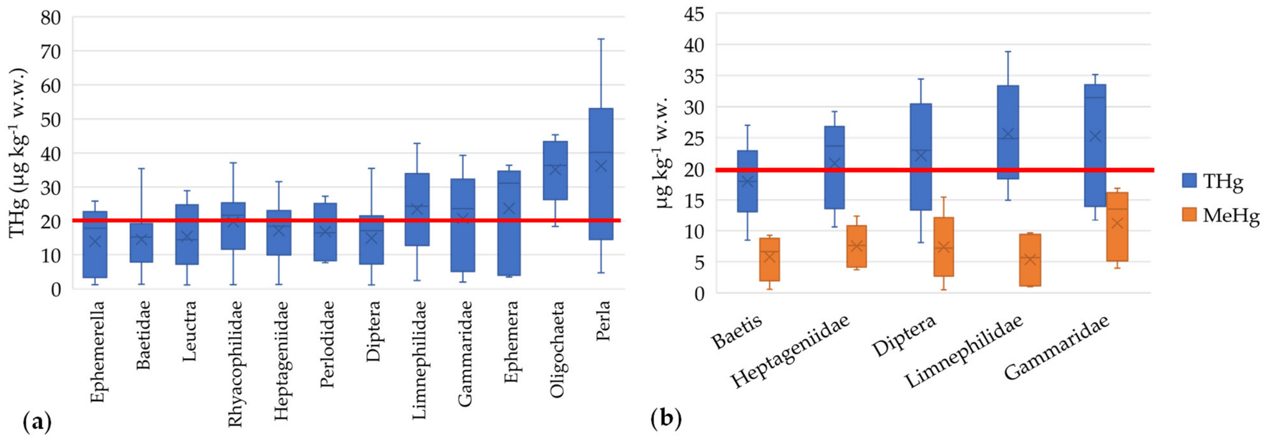

Figure S5. Box plot of total mercury and MeHg concentrations in benthic invertebrates collected in the Toce River reported to wet weight for comparison to the European Environmental Quality Standard for biota of 20 μg kg^{-1} w.w. as THg (red line): (**a**) THg considering all data; (**b**) THg and MeHg considering data collected in 2017.

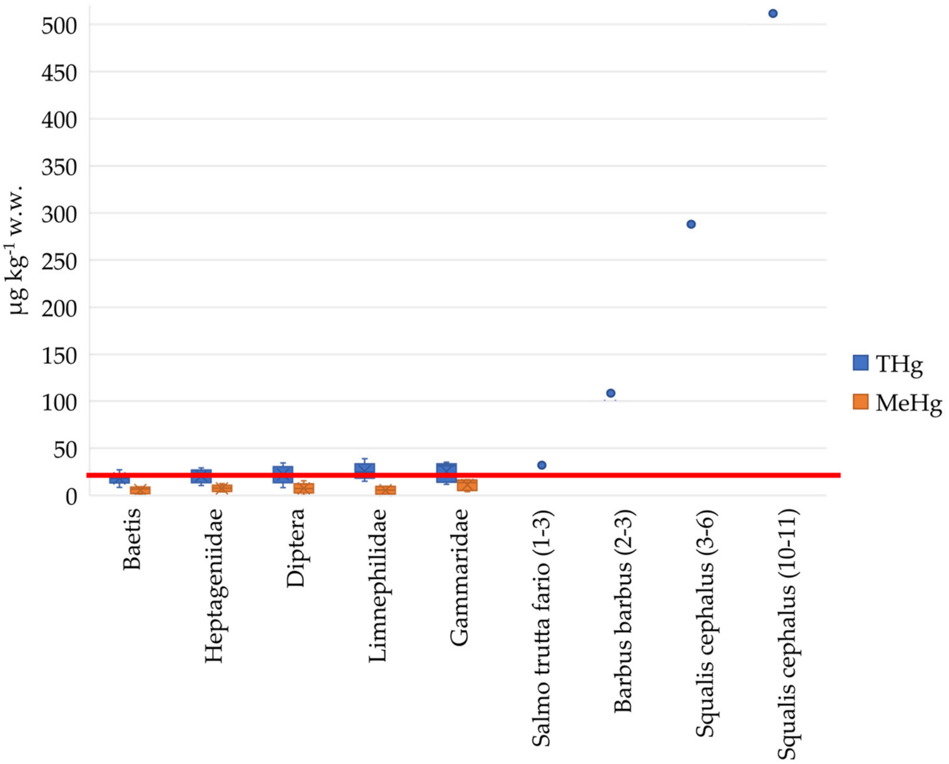

Figure S6. THg and MeHg concentrations in benthic invertebrates (box plots) and THg in fish (blue points) collected in the Toce River in 2017 and 2017-2019, respectively. Values for fish are obtained from analyses of pools of caudal fillets deriving from 2-6 specimens (in brackets, range of the fish age in years) [58,59]. The red line represents the European Environmental Quality Standard for biota (20 µg kg^{-1} w.w. as THg).
